# Inhaled Indocyanine Green Negative-contrast Fluorescence-guided Surgery for Pulmonary Metastasectomy

**DOI:** 10.1055/a-2780-3527

**Published:** 2026-01-24

**Authors:** Ángel Javier Gallego Fernández, Jose Andrés Moreno Delgado, Juan Francisco Navarro Pardo, Eloísa Diaz Moreno, Cristina Palomares Garzón, Ricardo Fernandez-Valadés

**Affiliations:** 1 Department of Pediatric Surgery, Hospital Universitario Virgen de las Nieves, Granada, Andalucía, Spain

**Keywords:** indocyanine green, thoracic pediatric surgery, inhaled

## Abstract

Indocyanine green fluorescence (ICG-F)-guided surgery has, in recent years, optimized the precision and safety of surgical procedures.

Although its applications are increasingly widespread, in most cases, the dye is administered intravenously.

We present a case of inhaled indocyanine green use for the identification and resection of pulmonary metastatic nodules.

A 12-year-old female patient with a history of sternal Ewing sarcoma with bone and pulmonary metastases was treated with chemotherapy, proton therapy, partial sternal and costal cartilage resection, and reconstruction with mesh and absorbable plates.

She later developed pulmonary relapse, with two subpleural metastases identified in segments 6 and 9 of the right lung. After initiating chemotherapy, thoracoscopic surgery was planned to resect the nodules.

Before surgery, nebulization of indocyanine green was performed via an endotracheal tube at 0.2 mg/kg using an inhalation chamber for 5 minutes at 6 liters per minute.

During surgery, fluorescence was observed in the insufflated lung parenchyma, allowing for clear differentiation of metastatic nodules from peripheral fibrotic or inflammatory tissue and enabling a safe wedge resection of both lesions.

The postoperative period was uneventful, and the patient is currently completing postoperative chemotherapy cycles.

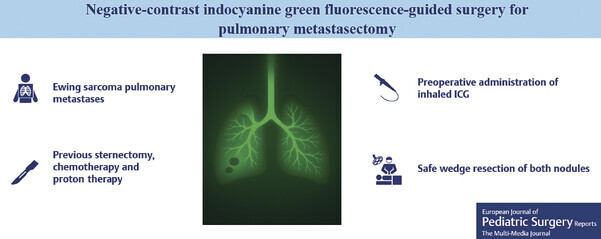

## Introduction

Indocyanine green (ICG) has experienced a remarkable resurgence in surgical fields due to its near-infrared fluorescence properties. Its strong affinity for plasma proteins and rapid biliary excretion make it an ideal optical agent for real-time visualization of anatomical structures, assessment of tissue perfusion, and lymphatic mapping. Although its use is well established in adults, particularly in hepatobiliary and oncologic surgery, its application in the pediatric population has expanded over the last decade, increasingly standardized across a range of procedures from tumor resections to complex reconstructions. 1

Although its indications are becoming more widespread, the dye is most commonly administered intravenously. In this context, the present article explores the clinical use of inhaled ICG for the identification and resection of pulmonary metastatic nodules.

## Case Description

**Video 1** Video demonstrating pulmonary parenchyma under ICG fluorescence versus metastatic nodules under negative-contrast fluorescence.


A 12-year-old girl with a history of sternal Ewing sarcoma with bone and pulmonary metastases was previously treated with chemotherapy, proton therapy, and surgery, including partial sternectomy with costal cartilage resection and reconstruction using resorbable polyglycolic acid and trimethylene carbonate mesh and plates.

**Fig. 1:**
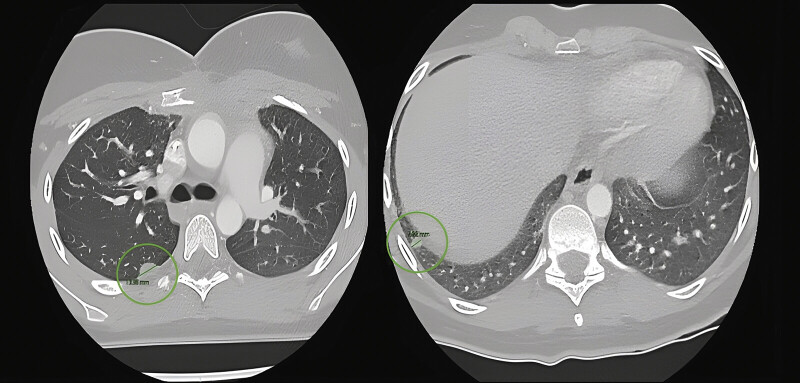
Preoperative CT scan showing metastatic nodules in segments 6 and 9.

At 10 months postoperatively, she presented with pulmonary recurrence, showing two subpleural metastases measuring 14 and 7 mm in segments 6 and 9 of the right lung, respectively. Following the initiation of chemotherapy, thoracoscopic surgery was proposed for nodule resection ( Fig. 1 ).

Prior to surgery, a dose of 0.2 mg/kg of ICG was nebulized via endotracheal tube using an inhalation chamber for 5 minutes at 6 liters per minute. Fluorescence was observed immediately upon completion of the inhalation period. The selected dose was based on previously reported pediatric experiences, 2 3 where 0.2 to 0.5 mg/kg provided optimal parenchymal fluorescence with minimal systemic exposure ( Video 1 ).

**Fig. 2:**
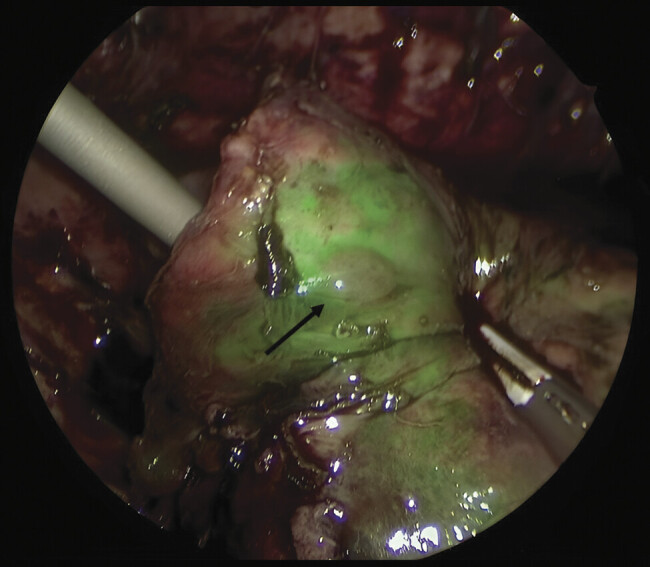
Metastatic pulmonary nodule in segment 9 under indocyanine green (ICG) negative-contrast fluorescence.

During the surgery, fluorescence of the ventilated lung parenchyma allowed clear differentiation of the metastatic nodules from surrounding fibrotic or inflammatory tissue and healthy lung, enabling a safe wedge resection of both lesions as negative-contrast areas, appearing dark against the fluorescent parenchyma ( Fig. 2 ).

The postoperative course was uneventful, and the patient is currently undergoing adjuvant chemotherapy without complications.

## Discussion

Thoracoscopic oncologic surgeries in pediatric patients are particularly challenging, not only due to the nature of the pathology but also because of their rarity compared to adult populations, presenting an additional difficulty for surgeons. Moreover, there is a growing trend toward minimally invasive approaches for congenital lung malformations, necessitating technological advances that enhance surgical safety in children.

Fluorescence-guided surgery using ICG has revolutionized intraoperative precision in various pediatric surgical specialties. Traditionally administered intravenously, ICG has proven to be a safe and effective method for anatomical visualization, perfusion assessment, and tumor resection. 1

However, a novel approach has recently emerged with the use of inhaled ICG as a surgical navigation agent in thoracoscopic procedures. This method, applied in thoracoscopic anatomical resections for congenital lung lesions, enables clear delineation between healthy tissue and lesions based on differences in ventilation. 2 The nebulized ICG is selectively absorbed by well-ventilated parenchyma, which fluoresces green under near-infrared light, while hypoventilated areas—such as lesions—remain dark. 3 This contrast facilitates precise and conservative resections, minimizing loss of functional lung tissue. 4

Furthermore, inhaled ICG has demonstrated itself as a safe, effective, and minimally invasive method for intraoperative localization of small pulmonary nodules. Unlike traditional techniques involving needle punctures, markers, or radiation exposure, this approach selectively targets ventilated lung tissue, creating a negative fluorescence contrast with hypoventilated lesions. It has shown particular value in localizing ground-glass opacities (GGOs), which are notoriously difficult to visualize or palpate intraoperatively. 5

This strategy significantly reduces localization time and improves detection rates, even helping avoid more extensive resections when nodules are not identifiable through conventional means. However, its use is limited to nodules located ≤1 cm from the pleural surface and may not be suitable for patients with severe pleural adhesions.

Additional limitations include the potential reduction of fluorescence intensity in patients with airway obstruction, ventilation–perfusion mismatch, or extensive atelectasis, as well as contraindications in those with known hypersensitivity to ICG or iodine, or severe respiratory compromise precluding adequate inhalation.

Only a few cases of inhaled ICG use have been reported in pediatric populations, mostly for congenital lung malformations 2 3 and nodule localization in adults. 4 5 Compared to these, our case is, to our knowledge, the first to describe its application for pediatric pulmonary metastasectomy, demonstrating feasibility and safety.

A more detailed comparison with previously published reports highlights key differences: in congenital lung malformations, inhaled ICG was primarily used to delineate anatomical segments rather than identify oncologic lesions 2 3 4 ; in adult series, its application focused on ground-glass opacities and peripheral nodules. 5 ^,6^ Unlike these, our case demonstrates its feasibility in a pediatric oncologic setting, where fibrotic changes from chemotherapy and previous thoracic surgery may further challenge intraoperative localization. This distinction underscores the uniqueness and potential applicability of the technique in complex oncologic scenarios.

## Conclusion

Despite being a novel technique, the ease of application, short learning curve, and reduction in surgical time and risks position inhaled ICG as a promising tool in precision pulmonary oncologic surgery. The use of inhaled ICG fluorescence enables clear differentiation between lung parenchyma and fibrotic or adherent tissues.

Additionally, this route of administration may also provide a safe approach for managing other congenital airway malformations, such as intralobar sequestrations. Future research should explore the optimal dosing, timing, and inhalation protocols for pediatric patients, as well as its comparative efficacy against intravenous ICG. Standardization of this technique may allow its broader adoption as a safe navigation tool for select oncologic and non-oncologic thoracoscopic procedures.

## Notes

Importance for the Pediatric Surgeon The use of inhaled indocyanine green fluorescence represents an advance in efficiency and safety for minimally invasive pediatric thoracic surgery.
